# The Catalytic Effect of Vanadium on Sorption Properties of MgH_2_-Based Nanocomposites Obtained Using Low Milling Time

**DOI:** 10.3390/ma16155480

**Published:** 2023-08-05

**Authors:** Zorana Sekulić, Jasmina Grbović Novaković, Bojana Babić, Milica Prvulović, Igor Milanović, Nikola Novaković, Dragan Rajnović, Nenad Filipović, Vanja Asanović

**Affiliations:** 1 Directorate for Energy and Energy Efficiency, Ministry of Capital Investments, The Government of Montenegro, Rimski trg 46, 81000 Podgorica, Montenegro; 2 Centre of Excellence for Renewable and Hydrogen Energy, Vinča Institute of Nuclear Sciences, National Institute of Republic of Serbia, University of Belgrade, Mike Petrovića Alasa 12-14, 11000 Belgrade, Serbia; bojana.babic@vin.bg.ac.rs (B.B.); igorm@vin.bg.ac.rs (I.M.);; 3 Faculty of Technical Sciences, University of Novi Sad, Trg Dositeja Obradovića 6, 21000 Novi Sad, Serbia; 4 Institute of Technical Sciences of Serbian Academy of Science and Arts, Knez Mihajlova 35, 11000 Belgrade, Serbia; 5 Faculty of Metallurgy and Technology, University of Montenegro, Cetinjski Put 2, 81000 Podgorica, Montenegro

**Keywords:** hydrogen storage, magnesium hydride, transition metals, additives, mechanochemistry

## Abstract

The effects of catalysis using vanadium as an additive (2 and 5 wt.%) in a high-energy ball mill on composite desorption properties were examined. The influence of microstructure on the dehydration temperature and hydrogen desorption kinetics was monitored. Morphological and microstructural studies of the synthesized sample were performed by X-ray diffraction (XRD), laser particle size distribution (PSD), and scanning electron microscopy (SEM) methods, while differential scanning calorimetry (DSC) determined thermal properties. To further access amorph species in the milling blend, the absorption spectra were obtained by FTIR-ATR analysis (Fourier transform infrared spectroscopy attenuated total reflection). The results show lower apparent activation energy (Eapp) and H_2_ desorption temperature are obtained for milling bland with 5 wt.% added vanadium. The best explanation of hydrogen desorption reaction shows the Avrami-Erofeev model for parameter n = 4. Since the obtained value of apparent activation energy is close to the Mg-H bond-breaking energy, one can conclude that breaking this bond would be the rate-limiting step of the process.

## 1. Introduction

Hydrogen as an energy vector represents great potential due to its high gravimetric density and low mass, as well as the fact that combustion does not emit harmful chemical byproducts. Hydrogen has the highest energy density per unit mass compared to any other fuel but a rather low energy density per unit volume. Further, hydrogen storage is a key technology for developing a hydrogen and fuel cell-based economy [1]. Metal hydrides as alternative hydrogen carriers have various performance parameters such as operating temperature, sorption kinetics, activation conditions, cyclic options, and equilibrium hydrogen pressure. These parameters can be improved or adjusted to meet the technical requirements of different applications [2]. Hydrogen in metal hydrides is chemically bonded, usually much stronger than the physical bonding present during hydrogen adsorption. Consequently, more energy is needed to release chemically bound hydrogen [3]. On the other hand, a stronger bond between hydrogen and metal hydride allows the storage of higher hydrogen densities even under ambient conditions [4]. Magnesium hydride is recognized as an excellent material for solid-state hydrogen storage due to its high gravimetric hydrogen capacity, good reversibility, and low cost. However, its practical application is limited by high thermodynamic stability and slow sorption kinetics [5]. To overcome the mentioned shortcomings, most of the research on magnesium hydride-based materials focuses on improving volumetric and gravimetric capacities, hydrogen absorption/desorption kinetics, and thermodynamics [1,2,3,4,5]. Mechanochemical modifications of the crystal structure of magnesium hydride and doping with the transition metals is a possible path for improving sorption properties. Doping with transition metals and their alloys is generally considered the simplest method to accelerate the sorption kinetics of MgH_2_. Most of the activities done in this field to the present day have been devoted to mechanochemical modifications using long milling times [1,2,3,4,5,6,7,8,9,10,11,12,13,14,15,16]. Sun et al. [4] compared the influence of transition metals in combination with carbon materials on the storage properties of hydrogen in MgH_2_. The desorption properties of the tested systems can be ranked as follows: Mg-Ti > Mg-Nb > Mg-Ni > Mg-V > Mg-Co > Mg-Mo. These composites can release hydrogen at temperatures below 225 °C, significantly lower than pure MgH_2_. Liang et al. [6] investigated the influence of 3d transition metals Ti, V, Mn, Fe, and Ni as additives on the sorption properties of MgH_2_, where powder nanocomposites were synthesized by a 20 h milling process. Desorption was fastest in the system MgH_2_-V, then MgH_2_-Ti, MgH_2_-Fe, MgH_2_-Ni, and MgH_2_-Mn at lower temperatures. On the other hand, the fastest absorption kinetics were determined for Mg-Ti, then Mg-V, Mg-Fe, Mg-Mn, and finally Mg-Ni. Of the investigated transition metals, V and Ti showed a better catalytic effect than Ni during hydrogen absorption and desorption. Composites with V or Ti as an additive showed fast desorption kinetics above 250 °C and absorption kinetics at temperatures below 30 °C. When examining the desorption properties of an MgH_2_-V system prepared by ball milling, Liang et al. [7] concluded that MgH_2_ −5 at.% V can desorb hydrogen at 200 °C and reabsorb hydrogen faster even at room temperature. It was found that the apparent activation energy of hydrogen desorption was reduced to 62 kJ/mol. Gasan et al. [8] investigated the influence of 5 wt.% of additives (V, Nb, and Ti) on the desorption temperature of hydrogen in MgH_2_. X-ray powder diffraction (XRD) results showed that adding vanadium powder significantly affected the transformation of Mg to MgO or hydride because the amount of MgO in the MgH_2_-V system was higher than in other systems. Scanning electron microscopy also showed a significant reduction in the particle size of the powder. The results obtained by differential scanning calorimetry showed that the addition of 5 wt.% additive reduces the desorption temperature of hydrogen in MgH_2_ by about 40–50 °C. Lu et al. [9] investigated the catalytic effect of two-dimensional (2D) vanadium nanoplates (VNS) on MgH_2_ for hydrogen storage purposes. It was found that the composite MgH_2_ + 7 wt.% VNS begins to release hydrogen at 187.2 °C, or at a temperature 152 °C lower than MgH_2_ without additives. Within 10 min at 300 °C, 6.3 wt.% of hydrogen was released from MgH_2_ + 7 wt.% of VNS composites. Additionally, a completely dehydrated sample can absorb hydrogen at room temperature under a hydrogen pressure of 3.2 MPa. Hanada et al. [10] investigated the catalytic effect of nanoparticles on 3d transition metals on hydrogen desorption in MgH_2_ prepared by ball milling. All MgH_2_ composites prepared by adding a small amount of Fe, Co, Ni, and Cu metal nanoparticles and ball milling for 2 h show much better hydrogen desorption than pure MgH_2_. The best properties were observed for an MgH_2_-based composite doped with 2 mol.% Ni nanoparticles and prepared by short-term (15 min) low-intensity ball milling (200 rpm). A large amount of hydrogen (~6.5 wt.%) was found to be desorbed in the temperature range from 150 °C to 250 °C by heating at a rate of 5 °C/min under a flow of He gas, practically without partial hydrogen pressure. According to DFT calculations done by Paskaš Mamula et al. [11], adding Fe, Ni, and Co will improve desorption kinetics due to the higher stability of Tm-H bonds. The final remark of numerous scientific papers on this topic is that transition metals have great potential in the choice of dopants from the aspect of improving sorption kinetics and reducing the activation energy of hydrogen desorption. On the other hand, most of the studies presented so far used similar high-energy mills and concerned milling at long times, from 1 to 25 h [1,2,3,4,5,6,7,8,9,10,12,13,14,15,16,17]. Further, as observed by Czujko et al. [18] there is not much difference in sorption kinetics of the mechanically modified MgH_2_ due to changes in the particle and crystallite size of the powder particles. Therefore, it is difficult to determine a parameter that influences kinetics.

In this paper, we have examined the structural transformations that occur in dopped MgH_2_ systems with different concentrations of vanadium at low milling times ranging from 15 to 45 min and correlated to the thermal behavior of the samples and kinetics.

## 2. Materials and Methods

Magnesium hydride (Langfang GreatAP Chemicals Co., Langfang, China, purity 98%) with the addition of vanadium (Merck, Rahway, NJ, USA, purity 99.99%) in different weight percentages (2 and 5 wt.%) was synthesized at different time intervals (15, 30, and 45 min) in an inert argon atmosphere to prevent oxidation of the sample, using BPR (Ball to Powder Ratio) ≈ 10:1. Mechanochemical synthesis was done in the SPEX Sample Mixer/Mill 5100 mill using stainless steel vial and balls, stainless steel jars, vials 1.2″, 1 gr balls, with a total of 100 mg sample amount [19]. The intensity of ball milling was 2500 rpm. The composition and parameters of milling are given in Table 1.

Structural characterization of material was performed using X-ray diffraction analysis (XRD) on a Rigaku Ultima IV diffractometer using a nickel filter and Cu Kα radiation (while wavelength was 0.1540 nm), in the 2θ range between 20 and 90°, at operating parameters of 40 kV and 40 mA, a step of 0.02°, the accumulative time of 5 s in every point and a silicon strip detector counter. The particle size distribution was analyzed using Mastersizer 2000, (Malvern Instruments Ltd., Malvern, UK) according to the standard procedure explained by Milanović et al. [20]. The desorption properties of the milled samples were measured by DSC apparatus SETARAM DSC131 using a heating rate of 10 °C/min, and temperature-programmed desorption (TPD) using homemade apparatus equipped with a mass spectrometer Extorr 300 at the same heating rate [21]. Iso-conventional kinetic methods and the Avrami-Erofeev kinetic model are described elsewhere [21].

## 3. Results

### 3.1. Microstructural and Morphological Characterization

**Figure 1:**
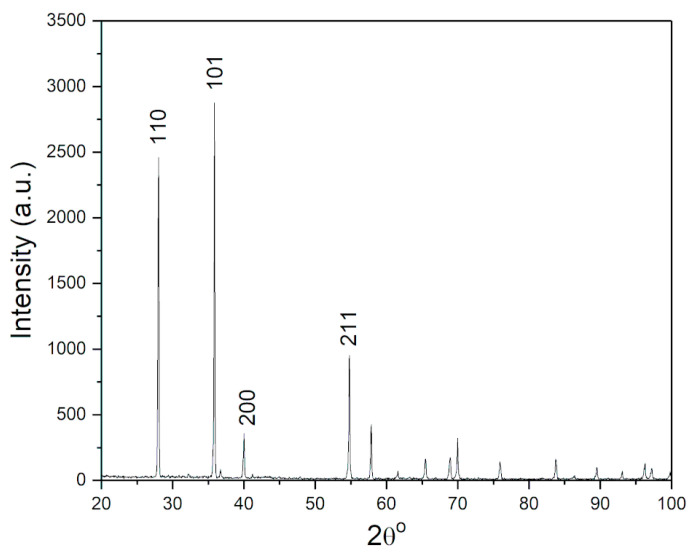
XRD pattern of as-received MgH_2_.

As shown in Figure 1, sharp diffraction maxima at positions 2θ = 27.63° (110), 35.62° (101), 39.48° (200), 54.21° (211), are characteristic of β-MgH_2_ with tetragonal structure, space group P_42_/mnm (No. 136) [22]. Low-intensity diffraction maxima at 2θ = 32.18° (100) and 36.63° (101) originating from metallic Mg as expected given the initial composition of the sample (98% purity). There are no peaks corresponding to the crystalline phases of Mg(OH)_2_ and MgO.

**Figure 2:**
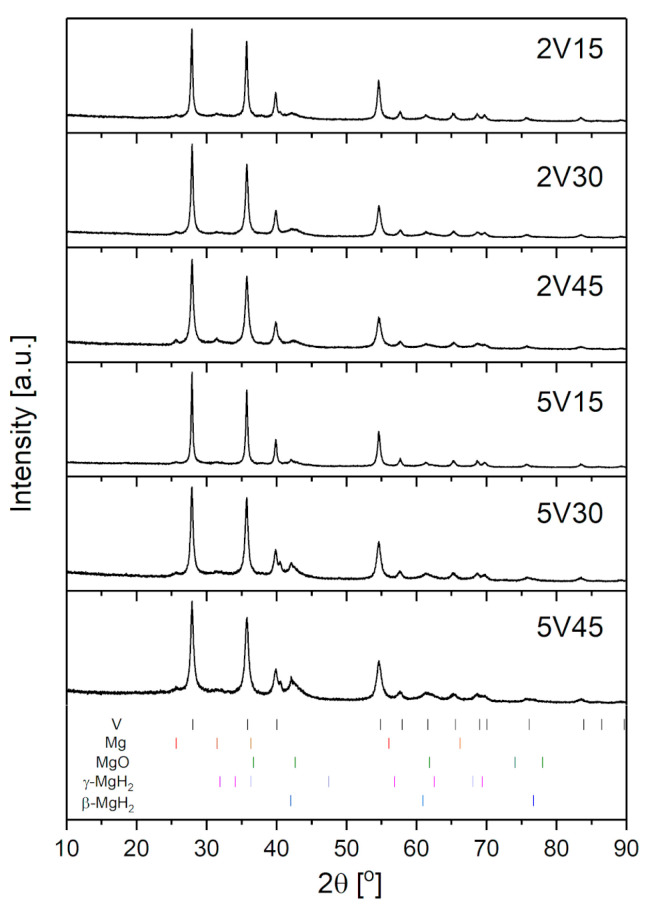
Diffractograms of milled MgH_2_-V composites obtained with different quantities of Vanadium (2 and 5 wt.%) and milled for 15–45 min.

Figure 2 shows diffractograms of all samples obtained using different milling times. The tetragonal β-MgH_2_ is the dominant phase, although a small amount of γ-MgH_2_ phase is present in samples even after 15 min of milling. The intensity of γ-MgH_2_ diffraction maxima increases with the increase of milling time since the amount of γ-MgH_2_ increases. The broadening of β-MgH_2_ phase diffraction maxima is visible, too, indicating a decrease in crystallite size. A similar was found by Varin et al. [23]. They have shown a spread of diffraction maxima in β-MgH_2_ that increases with increasing milling time. The authors associated this phenomenon with a decrease in crystallite size, which may be accompanied by the appearance of lattice micro-stress. Mixing with additives also introduces stress into the lattice, leading to peak broadening [12]. We expect similar behavior of samples milled with vanadium (see Table 2).

**Figure 3:**
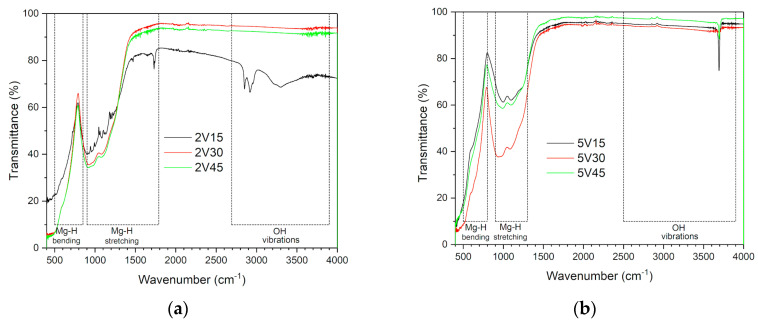
FTIR-ATR spectra of samples of MgH_2_ samples milled with 2 wt.% V (**a**) and 5 wt.% V (**b**) samples.

FTIR-ATR spectra for the samples milled with 2 and 5 wt.% of vanadium. are given in Figure 3a,b, respectively. Three regions are clearly distinguished: the first (500–800 cm^−1^) corresponds to Mg-H bending vibrations, the second (900–1300 cm^−1^) corresponds to Mg-H stretching vibrations [24], and for the 2V15 sample, and the third region (2500–3900 cm^−1^) corresponds to the observed OH group vibrations. For all three 5V samples, OH vibrations at 3670 cm^−1^ are observed. The largest changes are observed in the region 900 cm^−1^ FTIR-ATR spectra of MgH_2_ samples milled with 2 wt.% V (a) and 5 wt.% V (b) samples.

**Figure 4:**
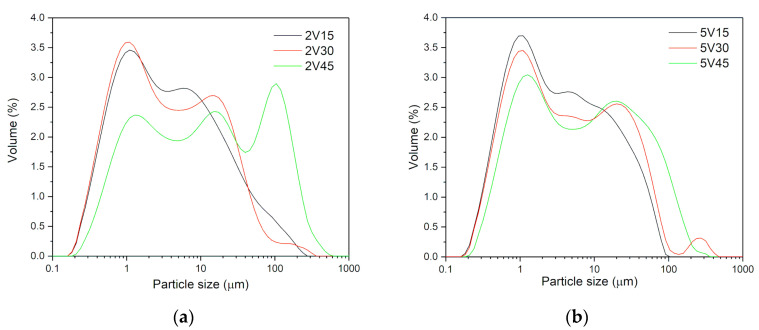
Particle size distribution of MgH_2_ samples milled with 2 wt.% V (**a**) and 5 wt.% V (**b**) samples.

Figure 4 shows the comparative distribution of particle sizes for all samples. As shown in Figure 4a, for all three milled blends with 2 wt.% of vanadium, about 30% of the particles have an average particle size of about 1 μm. Sample 2V15 has about 70% of particles with an average size of about 10 μm. As milling time increases, the average particle sizes increase due to agglomeration, and distribution becomes polymodal. This is followed by the specific surface area decrease of almost 50% for 2V and 5V samples. In Figure 4b the particle size distribution of samples milled with 5 wt.% of vanadium for different milling times: 15, 30, and 45 min (5V15, 5V30, and 5V45). The 5V15 sample has a particle size distribution in the range of 0.2–91.2 μm (about 75% of the sample is in the range of 0.2–10 μm, with an average value of 2.5 μm, while about 25% of the sample in the range of 10–91.2 μm, with the medium particle size of 39.3 μm).

The 5V30 sample is characterized by the widest distribution of particle sizes, polymodally distributed, where 65% of the sample consists of particles of 0.2–10 μm, with an average particle size of 2.3 μm. In the range of 10–100 μm, there is 30% of the sample, with an average particle size of 39.3 μm. The residue is outside this range, i.e., 100–416 μm, with an average particle size of 229 μm. The 5V45 sample has a similar polymodal particle size distribution as the 5V30 sample. Here, in the range of 0.2–10 μm, almost 60% of the sample is present, with an average particle size of about 2.62 μm; in the range of 10–100 μm there is about 37% of the sample, with an average particle size of 39.3 μm and the rest of the 5V45 sample powder consists of particles above 100 μm (from 100–316 μm) with an average particle size of 140 μm. Mechanical milling by adding 5 wt.% of the catalyst changes the shape of the distribution. However, as with the milled commercial material, the distribution is bimodal, except for the 5V30 sample, where the distribution is polymodal.

**Figure 5:**
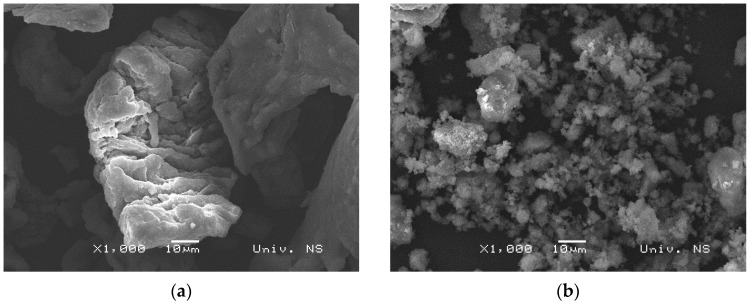
SEM micrographs of (**a**) commercial MgH_2_ powder and (**b**) MgH_2_ powder milled for 10 h.

The images obtained by SEM analysis (Figure 5) correlate with the particle size distribution analysis results. Commercial powder particles are irregular in shape with a layered structure, rough on the surface with a size above 100 μm, while powder particles MgH_2_ mechanically milled for 10 h are spongy in structure, with different sizes ranging from below 1 μm to 50 μm.

**Figure 6:**
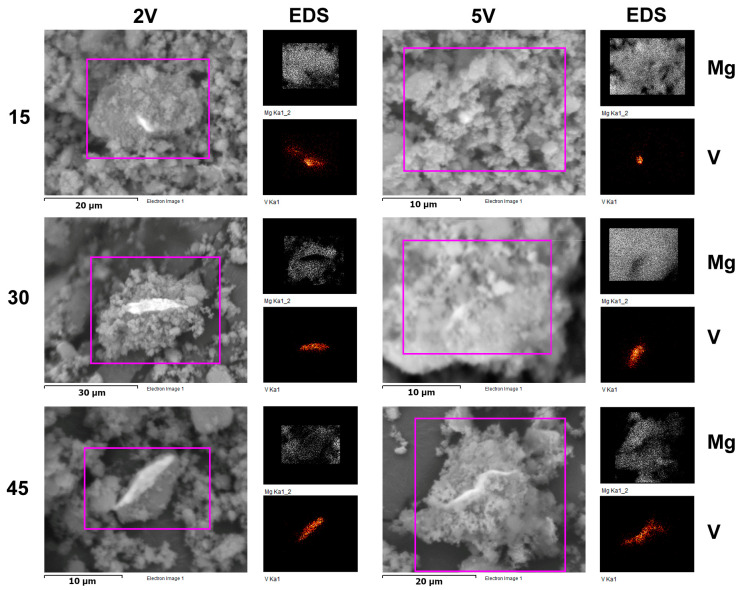
SEM micrographs and corresponding EDS Mg and V spectra of MgH_2_ samples milled with 2 wt.% V (2V) and 5 wt.% V (5V) samples.

SEM-EDS micrographs of composites MgH_2_-V show that vanadium layer particles are captured in MgH_2_ powder (Figure 6). EDS maps of vanadium and magnesium prove that the dispersion of vanadium particles is very weak in magnesium hydride powder, but the size of vanadium particles increases with the increase of milling time. As shown by Bassetti et al. [25], the microstructure and morphology of MgH_2_-Fe nanocomposites can be changed by turning the ball milling energy and catalyst concentration, thus affecting the kinetic features of the hydride decomposition. The microstructure–sorption properties interplay was and still is the subject of extensive research [23,26,27]. Czujko et al. first demonstrated that microstructure changes occur in the first few minutes of mechanical activation [18].

### 3.2. Thermal and Kinetic Characterization of Materials

**Figure 7:**
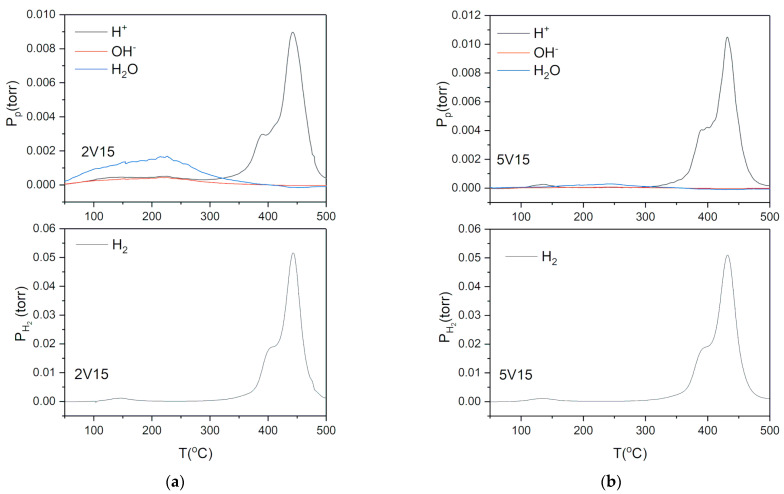
TPD profiles of MgH_2_ samples milled with 2 wt.% V (**a**) and 5 wt.% V (**b**) samples.

The TPD profiles of recombined hydrogen (H_2_), H^+^, OH^−^, and H_2_O from 2V15 (a) and 5V15 (b) samples have been shown in Figure 7. Multiple peaks at different temperatures suggest the existence of differently bonded hydrogen atoms in the samples [22]. If a low quantity of catalyst is added, there is a pronounced recombination of hydrogen and oxygen to form H_2_O, but also some OH^−^ ions are present too, possibly from the amorphous Mg(OH)_2_ phase [28], while in the 5V15 sample low-temperature peak originates from the recombination of H^+^ to H_2_ [29]. The existence of a low-temperature H_2_ peak is due to smaller hydride particles.

**Figure 8:**
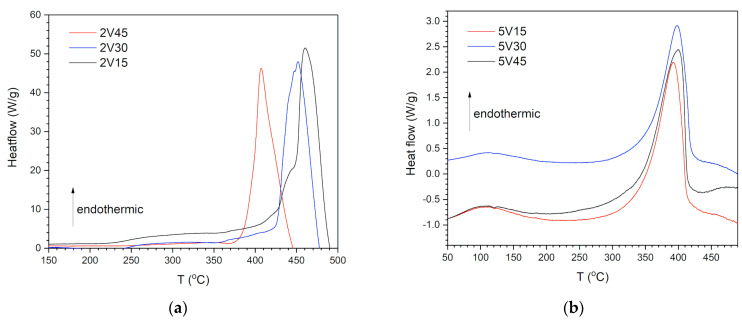
DSC curves MgH_2_ samples milled with 2 wt.% V (**a**) and 5 wt.% V (**b**) samples.

The DSC curve of commercial powder MgH_2_ shows the endothermic desorption maximum of hydrogen at 454 °C (high temperature, HT), a value comparable to the literature [30]. As indicated earlier, a sharp symmetric HT maximum originates from desorption from rutile-structure (β) MgH_2_. The maximum of very low intensity (intermediate temperature, IT) is observed at about 350 °C, which is also expected [29]. IT peak is a consequence of surface-bound OH groups. Suppose the sample is exposed to the atmosphere and oxidation for a long time, a third, low temperature (LT) maximum [24,25] may occur, which originates from OH groups and water. It is observed that the hydrogen release temperature increases with increasing milling time, meaning that shorter milling times have an improving effect on the desorption properties of MgH_2_ [18]. Figure 8a shows the thermograms obtained by DSC for 2V15, 2V30, and 2V45 samples. The endothermic desorption process in this composite significantly differs from the desorption from 5V samples. There is no desorption at low temperatures. Samples 2V15 and 2V30 composites are similar to pure milled hydride, with a wide maximum occurring at medium temperatures and an onset temperature of about 250 °C. For composites milled for 15 and 30 min, the maximum is shifted to temperatures above 454 °C. At temperatures of 450 °C and 460 °C, hydrogen is released from Mg(OH)_2_ due to the oxidation of MgH_2_ with oxygen from the air [31,32]. Figure 8b shows the thermograms obtained by DSC for 5V15, 5V30, and 5V45 samples. All three samples show a significantly different desorption profile than the commercial sample. The absence of desorption from medium temperatures is visible, but a pronounced LT peak appears at approximately 110 °C.

For sample 5V15, the HT maximum occurs at 392 °C and corresponds to the release of hydrogen from β − MgH_2_ (Table 3). The shift of the maximum by 62 °C is attributed to the reduction of the hydride particle size caused by mechanical milling. Similar behavior is shown by the other two composites milled for a longer time. The observed asymmetry of the DSC peaks could be explained by different PSDs and the presence of the gamma phase, as explained by Varin et al. [33].

Section 3.1 shows no significant changes in microstructural morphology upon short-term milling since similar microstructure and morphology were obtained for pure MgH_2_ [31]. This leads us to the conclusion that the addition of vanadium affects the thermal desorption of the material.

**Figure 9:**
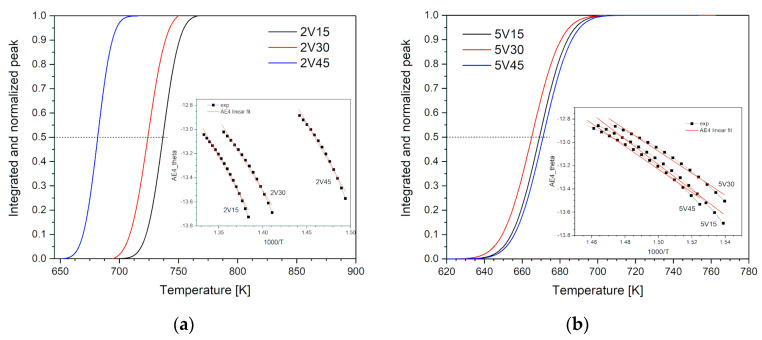
Temperature evolution of the reacted fraction (q) corresponding to MgH_2_-V decomposition, obtained by integration of HT-H_2_ peaks for mailing blends with 2 and 5 wt.% of vanadium and MgH_2_. Inserted figure: From ln g(θ) = f(1/T), the best fit of experimental data is obtained for nucleation. (**a**) sample with 2 wt.% of V, (**b**) sample with 5 wt.% of V.

To investigate the desorption process in detail, different models of solid-state kinetics were used as implemented in the code developed in our group. The rate-limiting step of the desorption reaction was determined using the iso-conversional kinetic method due to better accuracy of obtained apparent activation energies [21,34]. As shown in Table 3, showing the received MgH_2_ and milled one, for the same milling time [31], a decrease in apparent activation energies has been observed. It is obvious that the sorption kinetics is affected by material preparation because the reactivity of magnesium with hydrogen is strongly modified by changes in several surface parameters that govern the chemisorption, the dissociation of molecular hydrogen, and hydride nucleation [35,36]. Cui et al. [37] explained that desorption is affected significantly by the gas-solid interface. The first work by Isler [38] proposed that the reactivity of magnesium is determined by the free magnesium surface, while Vigeholm [39] indicates that surface nucleation and growth with a pressure-dependent concentration of nuclei is the mechanism of desorption. Therefore, the thermal decomposition of MgH_2_ is a heterogeneous reaction. If there is an additive in milling bland, the sorption kinetics is also affected by the reaction at the Mg-Tm metal surface [11], but also the dispersion of Tm must be considered when defining a mechanism of desorption [12]. As shown in Table 3, adding 5 wt.% of vanadium leads to a significant decrease in apparent activation energies and temperature, 78 kJ/mol and 392 °C. An explanation of the change of mechanism is given. This value of apparent activation energy is close to the Mg-H bond-breaking energy suggesting that the breaking of this bond would be the rate-limiting step of the process [40]. The kinetic analysis done by Perejon et al. [40]. on pure MgH_2_ shows that the reaction of desorption follows the first-order kinetics, equivalent to an Avrami-Erofeev kinetic model with a corresponding coefficient equal to 3, suggesting that the mechanism of tridimensional growth of nuclei previously formed (A3) if desorption is done under pressure. Similar is obtained by other authors [4,5,6,7,8,9,10,12,13,14,15,16]. The additive change mechanism of desorption to Avrami Erfeev with parameter 4 (A4) is shown in Figure 9. Random nucleation is observed in all systems where defects are introduced [34].

## 4. Conclusions

Using mechanochemical milling as a green synthesis method in an inert argon atmosphere, a magnesium hydride-vanadium composite was synthesized with a ball-to-powder mass ratio of 10:1, but different milling times from 15 to 45 min. As an additive, vanadium was added in 2 wt.% and 5 wt.%. The microstructure and morphology of the samples were examined and correlated with desorption properties monitored. The microstructure was monitored by X-ray diffraction analysis and infrared spectroscopy with Fourier transform, while morphology was examined by particle size distribution and scanning electron microscopy (SEM). The thermal behavior of milling blends was followed by TPD and DSC measurements. It was noticed that the presence of a dopant lowers the desorption temperature of hydrogen by several tens of degrees due to the catalytic action of vanadium. The best performance according to desorption temperature and apparent activation energy is expected for the 5V30 sample. Particle size distribution showed that even short milling times significantly reduce the particle size, which changes from a monomodal for as-received MgH_2_ to a polymodal distribution for milling blends milled for 30 min. As this distribution follows the particle size distribution of pure, milled MgH_2_, we can assume that the decrease in desorption temperature is due to the added catalyst and its distribution in the bulk of the material. It has also been observed that, depending on the catalyst concentration, the temperature maximum shifts to the left or right of the value characteristic of pure hydride. TPD and FTIR measurements demonstrate the existence of OH^–^ ions and H_2_O molecules. Those species give rise to intermediate and low-temperature desorption. Low-temperature desorption of H_2_ is noticed and can be ascribed to a lower particle size of MgH_2_. It is shown that short milling times correspond to apparent activation energies of 70 kJ/mol, comparable to results obtained using longer milling times. Also, adding V using high-energy ball milling does not drastically influence the desorption temperature. This implies that it is better to use shorter milling times.

## Figures and Tables

**Table 1:** The list of prepared samples and milling conditions.

Composition	wt.% V	Milling Time [min]	Name	
MgH_2_ + V	2	15	2V	2V15
		30		2V30
		45		2V45
	5	15	5V	5V15
		30		5V30
		34		5V45

**Table 2:** **Crystallite size** dependence on milling time and chemical composition.

V Content (wt.%)/Milling Time (s)	15	30	45
**0**	20	39	30
**2**	39	35	30
**5**	46	30	25

**Table 3:** Positions of low temperature (LT), intermediate temperature (IT), high temperature (HT), and very high temperature (VHT). DSC maxima and corresponding apparent activation energies (Eapp). Estimated errors are given in parentheses.

Sample	LT (°C)	Eapp (kJ/mol)	IT (°C)	Eapp (kJ/mol)	HT (°C)	Eapp (kJ/mol)	VHT (°C)	Eapp (kJ/mol)
5V15	111.4(9)	4.91(7)	371.4(7)	27.7(3)	396.12(6)	90(1)	469.9(6)	60.3(2)
5V30	114.7(4)	5.21(7)	368.1(6)	25.9(3)	392.01(4)	78(1)	452.4(7)	61.5(7)
5V45	115.0(6)	3.38(6)	371.5(6)	32.3(3)	398.03(4)	82(1)	-	-
2V15	420(2)	59.5(4)	437.9(2)	224(2)	463.8(2)	108.9(7)	-	-
2V30	-	-	436.82(8)	250(2)	451.22(7)	96.9(5)	-	-
2V45	-	-	-	-	408.6(1)	106.7(7)	428.7(3)	158(1)

## Data Availability

Data is contained within the article The data presented in this study are available in this article.
